# Supplementary material for: Modeling glioblastoma heterogeneity as a dynamic network of cell states
Source: Mol Syst Biol. 2021 Sep 16;17(9):e10105. doi: 10.15252/msb.202010105 (PMC8444284; doi:10.15252/msb.202010105)
Supplement: Supplementary file 6 — Source Data for Figure 5 [file MSB-17-e10105-s004.zip › Figure5A_sourcedata/GSEA_3071/nosigdb_stateV.GseaPreranked.1622795159954/FELDMAN_2020_S.html]

Details for gene set FELDMAN\_2020\_S[GSEA]

|  || Dataset | state43071 |
| Phenotype | NoPhenotypeAvailable |
| Upregulated in class | na\_pos |
| GeneSet | FELDMAN\_2020\_S |
| Enrichment Score (ES) | 0.62951213 |
| Normalized Enrichment Score (NES) | 3.5662904 |
| Nominal p-value | 0.0 |
| FDR q-value | 0.0 |
| FWER p-Value | 0.0 |
Table: GSEA Results Summary

  

Fig 1: Enrichment plot: FELDMAN\_2020\_S      
 Profile of the Running ES Score & Positions of GeneSet Members on the Rank Ordered List

  

| PROBE | GENE SYMBOL | GENE\_TITLE | RANK IN GENE LIST | RANK METRIC SCORE | RUNNING ES | CORE ENRICHMENT || 1 | ATAD2 |  |  | 2 | 2.214 | 0.0174 | Yes |
| 2 | ESCO2 |  |  | 3 | 2.193 | 0.0351 | Yes |
| 3 | FAM111B |  |  | 4 | 2.012 | 0.0513 | Yes |
| 4 | HMGB2 |  |  | 5 | 2.011 | 0.0675 | Yes |
| 5 | PCNA |  |  | 10 | 1.908 | 0.0821 | Yes |
| 6 | MCM4 |  |  | 17 | 1.815 | 0.0955 | Yes |
| 7 | DHFR |  |  | 18 | 1.795 | 0.1100 | Yes |
| 8 | TYMS |  |  | 30 | 1.683 | 0.1214 | Yes |
| 9 | CCNE2 |  |  | 32 | 1.664 | 0.1346 | Yes |
| 10 | RRM2 |  |  | 34 | 1.660 | 0.1478 | Yes |
| 11 | CENPU |  |  | 36 | 1.645 | 0.1608 | Yes |
| 12 | CDCA7L |  |  | 39 | 1.619 | 0.1735 | Yes |
| 13 | BRCA1 |  |  | 46 | 1.592 | 0.1851 | Yes |
| 14 | HELLS |  |  | 48 | 1.588 | 0.1977 | Yes |
| 15 | MCM3 |  |  | 49 | 1.586 | 0.2105 | Yes |
| 16 | GINS2 |  |  | 57 | 1.528 | 0.2214 | Yes |
| 17 | CDK1 |  |  | 69 | 1.488 | 0.2313 | Yes |
| 18 | SMC2 |  |  | 73 | 1.466 | 0.2425 | Yes |
| 19 | CHAF1A |  |  | 77 | 1.441 | 0.2535 | Yes |
| 20 | UBE2T |  |  | 79 | 1.432 | 0.2648 | Yes |
| 21 | FBXO5 |  |  | 82 | 1.422 | 0.2759 | Yes |
| 22 | CLSPN |  |  | 84 | 1.417 | 0.2871 | Yes |
| 23 | SCRG1 |  |  | 87 | 1.404 | 0.2980 | Yes |
| 24 | MCM7 |  |  | 88 | 1.400 | 0.3093 | Yes |
| 25 | MCM10 |  |  | 92 | 1.389 | 0.3199 | Yes |
| 26 | DEK |  |  | 94 | 1.384 | 0.3309 | Yes |
| 27 | CARHSP1 |  |  | 101 | 1.362 | 0.3407 | Yes |
| 28 | POLD3 |  |  | 111 | 1.340 | 0.3497 | Yes |
| 29 | CENPM |  |  | 114 | 1.337 | 0.3601 | Yes |
| 30 | FEN1 |  |  | 139 | 1.296 | 0.3659 | Yes |
| 31 | GMNN |  |  | 146 | 1.280 | 0.3750 | Yes |
| 32 | CENPK |  |  | 162 | 1.241 | 0.3821 | Yes |
| 33 | LMNB1 |  |  | 165 | 1.238 | 0.3917 | Yes |
| 34 | DNMT1 |  |  | 166 | 1.237 | 0.4016 | Yes |
| 35 | CDCA3 |  |  | 169 | 1.230 | 0.4112 | Yes |
| 36 | RFC4 |  |  | 171 | 1.227 | 0.4208 | Yes |
| 37 | NASP |  |  | 180 | 1.212 | 0.4291 | Yes |
| 38 | USP1 |  |  | 202 | 1.168 | 0.4344 | Yes |
| 39 | ZWINT |  |  | 204 | 1.166 | 0.4436 | Yes |
| 40 | ARL6IP1 |  |  | 212 | 1.158 | 0.4516 | Yes |
| 41 | CDC45 |  |  | 228 | 1.138 | 0.4578 | Yes |
| 42 | ASF1B |  |  | 237 | 1.130 | 0.4654 | Yes |
| 43 | RFC3 |  |  | 239 | 1.130 | 0.4743 | Yes |
| 44 | MCM5 |  |  | 243 | 1.127 | 0.4828 | Yes |
| 45 | HAUS8 |  |  | 273 | 1.089 | 0.4859 | Yes |
| 46 | BRCA2 |  |  | 274 | 1.089 | 0.4947 | Yes |
| 47 | MCM6 |  |  | 282 | 1.080 | 0.5020 | Yes |
| 48 | LYPD1 |  |  | 299 | 1.067 | 0.5075 | Yes |
| 49 | DUT |  |  | 314 | 1.050 | 0.5132 | Yes |
| 50 | CDC6 |  |  | 328 | 1.040 | 0.5191 | Yes |
| 51 | CDCA7 |  |  | 330 | 1.038 | 0.5273 | Yes |
| 52 | RFC2 |  |  | 337 | 1.025 | 0.5344 | Yes |
| 53 | DNAJC9 |  |  | 373 | 0.979 | 0.5355 | Yes |
| 54 | PSMC3IP |  |  | 382 | 0.968 | 0.5417 | Yes |
| 55 | NOVA1 |  |  | 393 | 0.957 | 0.5475 | Yes |
| 56 | RPA3 |  |  | 395 | 0.953 | 0.5550 | Yes |
| 57 | SLBP |  |  | 403 | 0.949 | 0.5613 | Yes |
| 58 | DLGAP5 |  |  | 418 | 0.936 | 0.5661 | Yes |
| 59 | MAD2L1 |  |  | 421 | 0.932 | 0.5732 | Yes |
| 60 | VRK1 |  |  | 445 | 0.911 | 0.5761 | Yes |
| 61 | ORC6 |  |  | 449 | 0.908 | 0.5828 | Yes |
| 62 | AKAP12 |  |  | 487 | 0.878 | 0.5827 | Yes |
| 63 | RAD51 |  |  | 522 | 0.851 | 0.5830 | Yes |
| 64 | TK1 |  |  | 591 | 0.806 | 0.5763 | Yes |
| 65 | MSH6 |  |  | 593 | 0.804 | 0.5826 | Yes |
| 66 | CDCA4 |  |  | 615 | 0.790 | 0.5849 | Yes |
| 67 | CENPA |  |  | 627 | 0.786 | 0.5891 | Yes |
| 68 | SIVA1 |  |  | 646 | 0.774 | 0.5918 | Yes |
| 69 | ARL6IP6 |  |  | 702 | 0.745 | 0.5872 | Yes |
| 70 | UNG |  |  | 703 | 0.745 | 0.5932 | Yes |
| 71 | SMC3 |  |  | 708 | 0.743 | 0.5984 | Yes |
| 72 | TFDP1 |  |  | 711 | 0.742 | 0.6040 | Yes |
| 73 | ATAD5 |  |  | 721 | 0.739 | 0.6082 | Yes |
| 74 | PGP |  |  | 727 | 0.732 | 0.6131 | Yes |
| 75 | TEX30 |  |  | 730 | 0.730 | 0.6186 | Yes |
| 76 | HAT1 |  |  | 741 | 0.727 | 0.6225 | Yes |
| 77 | AURKA |  |  | 761 | 0.718 | 0.6246 | Yes |
| 78 | TGIF1 |  |  | 840 | 0.683 | 0.6151 | Yes |
| 79 | H2AFZ |  |  | 851 | 0.677 | 0.6186 | Yes |
| 80 | RNASEH2A |  |  | 859 | 0.674 | 0.6226 | Yes |
| 81 | C19orf48 |  |  | 893 | 0.661 | 0.6216 | Yes |
| 82 | SRSF7 |  |  | 921 | 0.650 | 0.6216 | Yes |
| 83 | PTTG1 |  |  | 936 | 0.647 | 0.6241 | Yes |
| 84 | ZFAND6 |  |  | 958 | 0.638 | 0.6252 | Yes |
| 85 | TMEM97 |  |  | 963 | 0.636 | 0.6295 | Yes |
| 86 | RANBP1 |  |  | 999 | 0.620 | 0.6277 | No |
| 87 | MIS18A |  |  | 1023 | 0.614 | 0.6282 | No |
| 88 | SRSF2 |  |  | 1084 | 0.597 | 0.6214 | No |
| 89 | MSH2 |  |  | 1107 | 0.590 | 0.6219 | No |
| 90 | ANP32B |  |  | 1187 | 0.566 | 0.6112 | No |
| 91 | HNRNPF |  |  | 1216 | 0.559 | 0.6103 | No |
| 92 | EXOSC8 |  |  | 1230 | 0.553 | 0.6122 | No |
| 93 | RMI2 |  |  | 1261 | 0.542 | 0.6108 | No |
| 94 | CCNB2 |  |  | 1373 | 0.513 | 0.5934 | No |
| 95 | YWHAH |  |  | 1407 | 0.502 | 0.5911 | No |
| 96 | GGCT |  |  | 1414 | 0.501 | 0.5940 | No |
| 97 | EXOSC9 |  |  | 1472 | 0.487 | 0.5869 | No |
| 98 | POLR3K |  |  | 1476 | 0.487 | 0.5902 | No |
| 99 | HNRNPAB |  |  | 1541 | 0.471 | 0.5816 | No |
| 100 | DERA |  |  | 1749 | 0.428 | 0.5450 | No |
| 101 | DCTPP1 |  |  | 1796 | 0.418 | 0.5395 | No |
| 102 | PLAU |  |  | 1863 | 0.408 | 0.5300 | No |
| 103 | MEST |  |  | 1980 | 0.391 | 0.5107 | No |
| 104 | MALAT1 |  |  | 1988 | 0.391 | 0.5125 | No |
| 105 | MANF |  |  | 2023 | 0.384 | 0.5090 | No |
| 106 | PA2G4 |  |  | 2112 | 0.372 | 0.4950 | No |
| 107 | PPIF |  |  | 2478 | 0.325 | 0.4270 | No |
| 108 | RPA2 |  |  | 2570 | 0.314 | 0.4120 | No |
| 109 | TSC22D1 |  |  | 2978 | 0.272 | 0.3354 | No |
| 110 | HSPB11 |  |  | 3163 | 0.254 | 0.3019 | No |
| 111 | EEF1A1 |  |  | 3617 | -0.318 | 0.2168 | No |
| 112 | BAMBI |  |  | 3718 | -0.334 | 0.2002 | No |
| 113 | YPEL5 |  |  | 3745 | -0.339 | 0.1979 | No |
| 114 | ARL6IP5 |  |  | 3781 | -0.346 | 0.1939 | No |
| 115 | EIF2S1 |  |  | 3809 | -0.351 | 0.1915 | No |
| 116 | EEF2 |  |  | 3913 | -0.369 | 0.1745 | No |
| 117 | RPS27L |  |  | 3927 | -0.373 | 0.1750 | No |
| 118 | DDIT4 |  |  | 4182 | -0.432 | 0.1293 | No |
| 119 | LIMS1 |  |  | 4781 | -0.698 | 0.0193 | No |
| 120 | RAB13 |  |  | 4786 | -0.701 | 0.0242 | No |
| 121 | CALD1 |  |  | 4960 | -0.877 | -0.0022 | No |
| 122 | FTL |  |  | 5072 | -1.077 | -0.0150 | No |
| 123 | PHLDA2 |  |  | 5163 | -1.384 | -0.0213 | No |
| 124 | GJB2 |  |  | 5174 | -1.433 | -0.0117 | No |
| 125 | TPM1 |  |  | 5215 | -1.643 | -0.0062 | No |
| 126 | TAGLN |  |  | 5281 | -2.669 | 0.0027 | No |
Table: GSEA details [plain text format]

  

Fig 2: FELDMAN\_2020\_S: Random ES distribution      
 Gene set null distribution of ES for **FELDMAN\_2020\_S**
